# May

**Published:** 1890-05-03

**Authors:** 


"Hail, bounteous May, that dost inspire
Mirth, and youth, and warm desire;
Woods and groves are of thy dressing,
Hill and dale doth boast thy blessing."

The spring is now complete, and "merry May first early calls the morn." The winds have done their work, and the rains, although sometimes more than could be desired, do not saturate the earth beyond the power of the sun to dry it. The mornings, at least in the country, are clear, for "Zephyr and Aurora play." There are daily blue skies, although flecked with fleecy clouds, and, as Leigh Hunt observed, there are nights in which "the moon seems to be looking at the stars, like a young shepherdess at her flock." With loveliness the whole face of Nature turns from the iron clasp of winter to kiss the balmy lips of returning summer, and to welcome the bridal gifts of sun and shade. For

"Flowery May now from her green lap throws
The yellow cowslip and the pale primrose."

The meadows are thick with bright green grass, spangled with white daisies and yellow buttercups. Fruit trees put out their silver blossoms, and buds of various kind, both wild and cultivated, spring into life—for "March winds and April showers, bring forth May flowers." It was this universal springing up of life which probably originated Shakespeare's idea as expressed in the line, "In the May morning of his youth, ripe for exploits." Although there is an old saying, "Ne'er change a clout till May is out," we can yet with some degree of safety—always provided the season is an ordinary one—don lighter clothing than that worn in the depth of winter. Also lighter coloured clothing, especially for the fairer sex, will be more appropriate.

Persons who have gone abroad to avoid the English winter may now return with safety, for there is no climate in the world more suitable for the Anglo-Saxon, than the English summer of an ordinary year. And when the Anglo-Saxon goes abroad, he will do well to choose that country which most approaches in climatic characteristics an ordinary English summer.

The origin of the name May is doubtful. Some think the period was so designated in honour of the Roman senate, known as Maiores or Majories. Others derive May from Maya, the mother of Hermes; or from Mara, the mother of Mercury. However this is, May was held sacred to the ever bright Apollo, the son of Jove by Leto. From the 1st to the 3rd of May, among the Romans was the festival known as Floraria, sacred to the goddess of flowers and love—still a charming fête in Southern Europe, when all seek for Flora's favours. Even in northerly Britain it was formerly the custom for all ranks of people to go "a maying" early on the first of May. "The people rose at early dawn to go a maying." We read that in "the reigne" of Henry VI. the Alderman of London were on May Day at the Bishop of London's wood, "having there a worshipful dinner for themselves." It is also said that on the 1st of May "to Islington and Hogsdon runnes the streams of giddie people to eat cakes and creames." In London May Day was once as much observed as it is now neglected. The caricatures of may-poles, morris dancers, and Jacks-in-the-green, we now occasionally see got up—principally by boys—are but a faint echo of the May-day festival observed by our ancestors. Even the chimney-sweeps are not to the front as of yore, when, in consequence of the decrease of their work from the advent of summer "they seek to avail themselves of the liberality of festive meetings." For some time, however, May-poles were forbidden as "heathenish and idolatrous." In an old work, dated 1646, there occur the lines, "And harmless May-poles all are railed upon, as if they were the towers of Babylon." "Where the tall May-pole once o'erlooked the Strand," there now stands a church. There was once there a May-pole, one hundred feet in height, which was afterwards removed to Shooters Hill. On May 6th, it is stated that bird-catchers formerly first commenced to peer about the fields and thickets in search of different species of song-birds, for the purpose of netting, and training them for sale. It may be feared that bird catchers are not so particular in these days, and do not wait till the 6th of May to commence operations. On May 7th, Thunny fishing commenced. May 13th was the old May Day, when the ancient Druids used to light fires and sacrifice to Baal. At a place in Yorkshire a peculiar stone, called the "Druids' Stone," is shown. A visitor asked the young woman who acted as guide, "But who was the Druid, and does he ever come now?" "Well, I don't justly know," says the girl, "but feyther does"! On May 14th, on Whit-Sunday, it was usual in old catholic England to dramatize the descent of the Holy Ghost in the churches. Whit-Monday was always kept as a holiday, long before bank-holidays were instituted. Then "Kiss-in-the-Ring" was the favourite game. The hiring of farm servants is still continued in many parts of England on Whit Monday, although we believe the cudgel playing and the drinking of Whitsun ales to excess has rather gone out of fashion. In former days it was a triumph for a village belle to be chosen "Lady of the Ale." On May 18th, in 1664, Charles II. announced his royal will and purpose to continue the healing of his people for the evil during the month of May, and then cease till next Michaelmas; and they were not to come up in the interval and "so lose their labour." Unfortunately the power of healing the evil has departed from our kings of the present day! May 21st is Trinity Sunday. And May 24th is chiefly memorable because, in 1736, Jack Ketch, on returning from doing his duty at Tyburn (hanging people), robbed a woman of 4s. 6d., and the *Gentleman's Magazine* of the period! May 29th is noted as King Charles' Day, and it was long usual to decorate hats and horses heads with oak leaves on this anniversary, as some imagine, in commemoration of King Charles hiding himself in an oak tree, after his flight from Worcester. But this took place in September, not in May. May is not specially rich in saints days, but St. Dunstan's is mentioned. St. Dunstan was Archbishop of Canterbury in 988, and when at work on some sacred jewellery, being annoyed by Satan, he seized the devil with one of his tools. The legend has been given to us in these lines—

"St. Dunstan, as the story goes,
Once pull'd the devil by the nose
With red-hot tongs, which made him roar
That he was heard a mile or more."

Towards the end of May the beehives send their early swarms. The queen bee, having laid many eggs, is reduced to a slender shape, and becomes fitted for flight. It seems rather a pity human beings cannot get rid of surplus population by a similar easy exodus—without the queen, however. At this time also the glow worm begins to shine on her mossy couch. The male *lampyris noctiluca* does not emit a light, so perhaps, therefore, should not be so termed. But the light of the English glow-worm is not to be compared with some species of *elateridæ*, or fire-fly, several of which, it is stated, put into a bottle, afford illumination sufficient for the women of some parts of South America to see to carry on their ordinary work.

There are drawbacks, however, even to the month of May. Among the Romans it was considered unlucky to contract marriages in this month. At the present period it is not a time marked by many matrimonial alliances, would-be Benedicts appearing rather desirous of deferring the surrender of their liberty to the end of the season. Perhaps it is recollected, that although "Maids are May when they are maids," there is no guarantee what month they resemble afterwards! May is also the time when the May weed, or "stinking chamomile," "Pulls down both rye and wheat." Thomson sang, "Be gracious Heaven, for now laborious man has done his part." And not without reason. For, exceptionally, spring is held back by bitter winds and frosts. The bloom in England sometimes hovers in nightly peril till past the middle of May. The fruit crop trembles in the hazard of a single night of frost. After a bright May day and a cold starry night, the outside of the bud may look fair, but the inside is nipped and destroyed, and will not become fruit. On the continent, especially in Germany, it may be worse. The 7th of May is the date of the "Icy Saints," when a cold blast has been known to flow over a hundred leagues and smite all. But let us not be dissatisfied with our May, for we may go further and fare worse.

